# Use of complementary and integrative health in Finland: a cross-sectional survey

**DOI:** 10.1186/s12906-023-04088-4

**Published:** 2023-08-04

**Authors:** Maija Pyykkönen, Pauliina Aarva, Salla Ahola, Matti Pasanen, Kaija Helin

**Affiliations:** 1 Socon – Social and Health Consultants Ltd, Tampere, Finland; 2 Faculty of Social Sciences, Tampere University, Tampere, Finland; 3 Faculty of Management and Business, Tampere University, Tampere, Finland; 4 Consultant in Statistics, Tampere, Finland; 5 Finnish Society for Integrative Medicine, Helsinki, Finland

**Keywords:** Complementary and integrative health (CIH), Complementary and alternative medicine (CAM), CIH use, Prevalence, Finland

## Abstract

**Background:**

Population based studies have shown large differences in the estimated prevalence of complementary and integrative health (CIH) usage between studies. This is in part due to there being no golden standard definition for CIH. In Finland, an updated and internationally comparable study on the prevalence of CIH usage is needed. In the present study, a modified Finnish version of the International Questionnaire to Measure Use of Complementary and Alternative Medicine (I-CAM-QFI) was utilised to examine prevalence of use of different CIH modalities and their experienced helpfulness in the general Finnish population.

**Methods:**

Respondents aged 16 and above were invited to take part in this descriptive cross-sectional study through an online panel in December 2022. The usage of CIH and the experienced helpfulness were calculated with SPSS (v28) as the proportion of users per each modality. The data were weighted based on gender, age and place of residence.

**Results:**

A total of 3244 respondents completed the survey. CIH was used by 51.1% (95%CI: 49.4–52.8) of the respondents in the 12 months prior to the survey. Self-help practices were the most used category of CIH (28.8%; 95%CI: 27.3–30.4). The prevalence of usage of CIH natural remedies excluding vitamins and minerals was 27.0% (95%CI: 25.5–28.6). CIH providers were visited by 20.4% of the respondents (95%CI: 19.0–21.8). Getting help for a long-term illness or improvement of well-being were often mentioned as the most important reason for the use of different CIH modalities. CIH was generally used more by women compared to men. The large majority found the modalities they used helpful.

**Conclusions:**

The results increase current understanding on CIH usage in Finland. As the majority of users experience CIH as helpful, there is a need to study CIH in the context of public health policies. The estimates of CIH usage are highly dependent on what is considered as CIH, and this should be paid attention to in future studies.

**Supplementary Information:**

The online version contains supplementary material available at 10.1186/s12906-023-04088-4.

## Introduction

Complementary and integrative health (CIH) comprises a vast range of provider-led procedures (e.g. chiropractic and bone setting), natural remedies (e.g. herbal medicine and nutritional supplements) and self-help practices (e.g. meditation and yoga), that are offered along with or outside of conventional healthcare [1]. CIH is known to be used worldwide, and the prevalence of its usage has been studied in many countries [2–8]. CIH has been recognised as a significant public health issue by the World Health Organisation [1, 9].

Population based studies show large differences in the estimated prevalence of CIH usage between countries, estimates ranging between 10–40% [4] and 24–71.3% [10]. This is also evident in studies conducted within the same areas across time [5, 11]. This variability in the estimates of self-reported CIH usage is known to be in part dependent on the used definition of CIH, as no golden standard definition currently exists. The terms complementary, alternative, traditional and natural therapies and products have all been used in describing three main CIH modalities: the services offered by CIH providers and therapists, natural remedies and self-help practices [4, 12–15]. In this article we use the term CIH, as some CIH modalities included in the study are integrated into the healthcare system in Finland. Additionally, as there is no standard definition in the international health research field on the topic, terminology regarding CIH is multifaceted [15].

In order to optimise comparisons made between studies, a unified survey tool, the International Complementary and Alternative Medicine Questionnaire (I-CAM-Q)[16], was developed. I-CAM-Q has been translated to multiple languages and used in international research on CIH usage for example in Europe [17, 18], South America [19], and Asia [20]. Recently, Kristoffersen et al. [5] facilitated the Norwegian translation of the questionnaire (I-CAM-QN). The estimated prevalence of overall CIH usage in the general Norwegian population was 62.2% [5], a higher estimate compared to earlier studies conducted in Norway or elsewhere in Europe [5, 11]. These differences were suggested to be related to differences in methodology, for example the ways in which the survey questions were formulated.

CIH has been researched in Finland starting in the second half of the twentieth century [21–24], and recently interest has increased in investigating the prevalence of CIH usage in the general population. An international comparison study based on data from the 2014 European Social Survey (ESS) conducted by Kemppainen et al. [4] showed a prevalence of 35.3% for CIH usage within 12 months prior to the survey in Finland.

A study by Vuolanto et al. [25] examined the usage of CIH in Finland from data collected in 2008 and 2018. In addition, the Finnish Medical Association (FMA) analysed data on Finnish CIH usage collected in 2021 [26, 27]. The highest prevalence was found in the usage of natural remedies, vitamins and dietary supplements as well as for acupuncture, chiropractic and bone setting. The questions were formed to cover lifetime usage of CIH. Furthermore, Vuolanto et al. [25] included both self and family history of use in their prevalence estimates, complicating comparisons to studies focusing on individual usage of CIH. However, only 17.4% of the participants surveyed in 2018 informed that neither they nor their family members had ever used any CIH modalities, indicating that a large majority of the Finnish population has at least at some point in their lives used CIH [25]. This knowledge increases the need for a more detailed information and internationally comparable assessment of CIH usage in Finland.

Current Finnish healthcare regulations do not address CIH providers, unlike legislation in other Nordic countries of Sweden [28] and Norway [29]. In Finland, qualified chiropractors, osteopaths and naprapaths have been incorporated into the national health practitioner registry since 1998. Despite the official status of these providers they are widely considered as CIH providers in Finland, as evidenced by a recent survey by the Finnish Medical Association [26]. Furthermore, trained conventional massage therapists, but not other types of massage therapists, are included in the aforementioned official registry for health professionals [30]. Some CIH practitioners are self-regulated through voluntary membership of professional associations, and there are separate regulations in place for natural remedies [30–33]. Furthermore, a number of modalities perceived as CIH are included in some current best practice guidelines for physicians, for example acupuncture has been found effective in treatment of pain in knee or hip osteoarthritis [34]. Majority of CIH modalities fall outside the national healthcare system in Finland, this possibly resulting in increased accessibility divide between different socioeconomic groups [4].

There is a need to investigate the prevalence of CIH usage in Finland with the implementation of an internationally recognised questionnaire, such as I-CAM-Q. Furthermore, it is paramount to better understand the factors associated with CIH usage in moving forward evidence based policy making related to health system development and legislation. The aim of this study is to deepen and detail the understanding on the prevalence of CIH usage in the general population of Finland and to provide internationally comparable information, e.g. with other Nordic countries on CIH use. We expect that the results will be useful for possible future public health policy making in regards to CIH.

## Methods

### Sampling and recruitment

The data were collected from the commercial probability based online panel consisting of individuals aged 15 and above. The total size of the Kantar Public online panel is about 30 000 persons. Panel participants are continuously recruited to ensure that the quota samples drawn from it would be demographically similar to national populations. The quotas are set based on national census data [35]. In Finland internet coverage is fairly high, and 93% of the population aged 16–89 have used the internet during the past 3 months [36], making an online panel a feasible means for collecting data.

The survey was in Finnish. The questionnaire was sent by email to 11,507 panellists aged 16 and above living in mainland Finland (excluding Åland) between 2nd and 19th of December 2022 with several reminder messages. A link within the email directed the respondent to the survey platform maintained by the service provider Kantar Public. Prevention of repeated responses during data collection was ensured by Kantar Public by allowing each respondent to complete the survey only once. The original aimed total number of respondents was 3000. A total of 3244 responses were received, resulting in a response rate of 28,19%. All of the respondents were of the age of 16 or above. Participation in the survey was voluntary and panellists received a small compensation in the form of points for their participation in the surveys, which could be used to purchase goods. The data including panellists’ personal information was stored by Kantar Public. Confidentiality and anonymity was ensured by the data being fully anonymised and personal background information (name, contact details) removed, and this was done prior to the research group gaining access to the data. Only the researchers contributing to this study and named personnel of Kantar Public had access to the data. The data was stored on password protected network drive and computers during the research project. The data will be archived in the Finnish Social Science Data Archive (FSD) hosted by Tampere University after the research project has been completed.

### Questionnaire

A modified Finnish version of the Norwegian questionnaire I-CAM-QN [5] based on the original I-CAM-Q [16] was used as the survey tool (I-CAM-QFI; included in the Supplementary material). The I-CAM-QN was translated from Norwegian into Finnish by the research group. A professional translator back translated the Finnish translation of the I-CAM-QN into Norwegian for quality check of the original translation. Additionally, the original English I-CAM-Q was translated into Finnish by the research team, and back translated into English by a professional translator. Last, the two Finnish translations were compared, and the Finnish version was finalised. The questionnaire was piloted with 57 respondents by Kantar Public. The small-scale pilot was to test the functionality of the survey questionnaire and optimise the included questions. The questionnaire was then revised by the research group.

The survey includes three main categories: services offered by providers, natural remedies and self-help practices. All modalities included in the survey are listed in Tables 4, 5, 6 and 7. Parts of the original questionnaire (I-CAM-Q) were modified to fit the current view of what modalities are defined as CIH in Finland. For the included CIH providers, following changes were made compared to I-CAM-QN: bone setter, aromatherapist, art therapist, hypnotherapist, traditional Chinese medicine (TCM) practitioner, anthroposophic therapist and ayurvedic practitioner were added to the list of surveyed CIH providers. These additions were made based on previous CIH research in Finland indicating that these CIH modalities are used in Finland [25, 26].

Healer and kinesiologist were removed from the list of CIH providers. In Finland, the word “healer” is not used, and instead the more general term is “folk healer” [37]. However, the modality of a “folk healer” was not included into the study, as the list of CIH providers utilised in our study at least partly covers the providers possibly perceived as folk healers, e.g., a cuppist or a bone setter. The modality of an energy healer is a more modern and more specific term for a more general term of a folk healer, and was thus included into the list of CIH providers. Additionally, kinesiologist is not a common CIH provider in Finland. Neither kinesiologist nor healer were included in the most recent CIH-surveys conducted in Finland [25, 26].

The “massage therapy” category used in I-CAM-QN was separated into two separate classes: traditional or conventional massage therapist and other types of massage therapist. Lightning process was removed from the list of CIH self-help practices based on its absence on the most recent CIH-surveys done in Finland [25, 26] and a recent Norwegian study using the I-CAM-Q [5] not having any respondents who had used it in the last 12 months. Mindfulness and meditation were combined into the same category, as were Tai Chi and Qigong. Sauna, art and nature were added to the list of self-help practices. No changes were made to the list of natural remedies used in the survey.

The order of the modalities presented for visits to providers and for use of self-help practices was randomised to avoid the possible influence of the presentation order on responses. The full survey used in the current study included additional questions to the I-CAM-QFI, results of which are presented in other publications.

### Measures

#### Measures of personal characteristics

In this study gender, age and place of residence were asked in the beginning of the survey. All other background variables (incl. education, personal and household income) were asked in a separate Kantar Public survey and updated annually for each panellist.

Information on household yearly income was collected with the following categories (20.000€ and below; 20.001–35.000€; 35.001–50.000€; 50.001–85.000€; 85.001–100.000€; 100.001€ and above). These were merged into three final categories for the final analysis: low (35.000€ and below), middle (35.001–50.000€), and high (50.001€ and above).

Education was collected by using six levels based on the highest form of obtained formal education: primary and lower secondary school, vocational upper secondary education, general upper secondary education, vocational education, Bachelor’s degree from university or university of applied sciences (or comparable higher vocational degree) and Master’s degree or higher from university or university of applied sciences. Further, these were combined into four categories (primary and lower secondary school; upper secondary education and vocational college education; Bachelor’s degree or higher vocational degree; Master’s degree or higher). It should be noted that the exact years of formal education might slightly differ between individuals in each category. This is in part due to degrees of higher education not having a fixed period of study in Finland, for example the completion duration of a bachelor’s degree typically varying between 3.5 and 4.5 years [38]. Additionally, part of the respondents had completed primary school before or during the primary school reform in Finland (1972–1977), which might have influenced the exact years of schooling.

Age was calculated based on year of birth. Age in years was merged into the same three categories as in the study by Kristoffersen et al. [5], and the categories were used in the final analysis (16–29; 30–59; 60 and above). Gender of the respondents was assessed via a categorical question (female; male). Residency was assessed by using the four designated NUTS (Nomenclature of Territorial Units for Statistics) level 2 areas of Finland: Helsinki-Uusimaa, South Finland, West Finland, North and East Finland.

#### CIH therapies by CIH providers

Visits to CIH providers were assessed by respondents indicating which CIH providers they had visited in the 12 months prior to taking part in the survey. A list of CIH providers was presented, including response options of “none of the above” and “I do not know/want to answer”. There was additionally a response option (“Other, what?”) for any CIH providers not included in the list with an open field to specify the CIH providers. The included providers surveyed are represented in Table 4. Three additional questions were included per each CIH provider visited: number of visits to the CIH provider in the last 3 months, the most important reason for the latest visit (acute condition lasting for less than one month; a long-term condition lasting for longer than one month or a related symptom treatment; improving well-being; other reason; I do not know), and if the modality was regarded as helpful (very helpful; somewhat helpful; not helpful; I do not know). We chose to use the term “provider” as it was used in the original English version of the I-CAM-Q [16].

Visits to non-CIH providers, including physician and conventional massage therapist, were assessed in the same list with CIH providers. As with CIH providers, if the respondent reported visiting a physician or a massage therapist in the 12 months prior to taking the survey, the same three additional questions were asked: number of visits in the last 3 months, the most important reason of the latest visit and whether visiting was considered helpful.

#### Natural remedies

Usage of natural remedies, i.e. herbal medicine and dietary supplements, within the 12 months prior to taking part in the survey was similarly assessed with a list, in which respondents could indicate to have used certain herbal remedies and supplements. The list included options for “other”, “none of the above” and “I do not know/want to answer”. The included natural remedies are represented in Table 5.

Even as many vitamins and minerals are used outside the healthcare system, some of them (for example multivitamins and calcium) may be used as part of conventional care in Finland and are thus not considered CIH in Finland. As the usage of specific vitamins and minerals was not assessed, we excluded the category “vitamins and minerals” from the calculations of usage of CIH natural remedies, and further from over-all CIH use.

#### Self-help practices

The assessment of CIH self-help practices included a list of self-help practices in which the respondent could indicate the modalities used in the 12 months prior to taking part in the survey. There was also an open-ended option “Other, what?” for any CIH self-help practices not mentioned in the list. Additionally, the list included items “none of the above” and “I do now know/want to answer”. The included CIH self-help practices are represented in Table 6. Respondents answered to three additional questions per each self-help practice they had used: number of times they had used the practice in the last 3 months, the most important reason of the last time they used the practice (acute condition lasting for less than one month; a long-term condition or illness lasting for longer than one month or a related symptom treatment; improving well-being; other reason; I do not know) and if they regarded the practice as helpful (very helpful; somewhat helpful; not helpful; I do not now know).

Usage of prayer for one’s health, sauna, art and nature as forms of self-help practices were included in the list of surveyed self-help modalities (Table 7). Same follow-up questions as for CIH self-help modalities were presented for respondents who had used any of the modalities in the 12 months prior to taking the survey.

#### Over-all use of CIH

Over-all CIH use was measured in the total number of CIH users. CIH users included respondents who reported the usage of at least one modality of CIH within the 12 months prior to taking part in the survey from the three CIH categories: CIH providers (not including traditional/conventional massage therapist), natural remedies (not including vitamins and minerals) and self-help practices (not including prayer for one’s health, sauna, art, nature and the “other” category). All modalities included in the definition are listed in Table 1.

**Table 1:** CIH modalities included into the definition of CIH user ^a^ in our study

CIH providers and therapists	CIH Natural remedies ^b^	CIH self-help practices
Chiropractor	Herbs and herbal medicine	Meditation and mindfulness
Homeopath	Homoeopathic remedies	Yoga
Acupuncturist	Other supplements (not vitamins and minerals)	Tai Chi and Qigong
Phytotherapist	Other	Relaxation techniques
Bone setter		Visualisation
Energy healer		Attending traditional healing ceremonies
Reflexologist		NLP ^c^
Aromatherapist		
Massage therapist (other, non-conv.)		
Naprapath		
Osteopath		
Art therapist		
Cuppist		
Hypnotherapist		
TCM practitioner ^c^		
Anthroposophic therapist		
Ayurvedic practitioner		
Other ^d^		

^a^ Using one or more of these modalities at least once in the 12 months preceding the survey

^b^ Vitamins and minerals were excluded

^c^ TCM = Traditional Chinese Medicine, NLP = Neurolinguistic Programming

^d^ The open ended answers coded as CIH (*n* = 22) were included

This definition was chosen in order to follow the definition used by Kristoffersen et al. [5] as closely as possible in order to improve comparability between the studies. In their study, they included visits to CIH providers, usage of natural remedies and CIH self-help modalities. There were some differences in both the modalities included between our studies, and in what constitutes CIH both in Norway and Finland.

In the list of possible CIH providers, an open ended answer option (“Other, what?”) was presented for respondents to name the providers not present in the original list. The answers (*n* = 199) were coded by the research team into two categories: CIH and not CIH. Only the answers coded as CIH (*n* = 22) were included in the final analysis for the “other” category. These answers included: craniosacral therapy, energy healer, erotic massage, folk healer, Gua Sha massage, hot stone massage, Indian head massage, light therapy, LPG therapy, lymph massage, massage chair, Neurosonic treatment, nutritionist, personal trainer, Shiatsu massage, spiritual healer, sports massage, Thai massage and Trager therapy. The rest of the answers consisted of therapists and providers regarded as part of the conventional healthcare system in Finland and were coded as not CIH (*n* = 177).

Similar open-ended answer option (“Other, what?”) was presented in the list of possible self-help practices. Out of the answers (*n* = 130), half (*n* = 65) mentioned some form of physical exercise, such as walking, strength training at the gym or pilates. However, as the scope of the answers was too broad to be analysed in this study, the “Other” category of self-help practices was not coded further and consequently not included in the analysis of overall use of CIH.

#### Intention to use

Intention to use CIH in the future was assessed with a question “Do you intend to use any complementary treatments in the future?”. Respondents were presented with answer options “Yes”, “Probably yes”, “Probably no”, “No” and “I do not know/I do not want to answer”. For the final analysis, the answer options were grouped into three categories (“Yes or probably yes”; “No or probably no”; “I do not know/I do not want to answer”).

#### The effects of Covid-19 on CIH usage

The self-reported effects of Covid-19 pandemic on CIH usage were assessed with two questions, one in relation to visits to CIH providers and the other in relation to usage of natural remedies and self-help practices. The answer options for both questions were “Decreased a lot”, “Somewhat decreased”, “Neither decreased nor increased”, “Somewhat increased”, “Increased a lot” and “I do not know/I do not want to answer”. In the final analysis, these answer options were merged into four categories for both separate questions (“Decreased”; “No change”; “Increased”; “I do not know/I do not want to answer”).

### Statistics

The usage of CIH and the experienced helpfulness were described as the proportion of users per each modality. The 95% confidence intervals (CI) were calculated for the proportion of users of CIH providers, CIH natural remedies, CIH self-help practices and over-all CIH usage in the last 12 months. For 3 month use the data was described by using both mean and median due to distributions of some modalities being highly skewed.

Data were weighted based on age, gender and region of residency in order to better represent the Finnish population. All results reported in the Results-section (see below) are based on weighted data. The data were analysed using IBM SPSS Statistics (version 28).

## Results

### Basic respondent characteristics

Basic respondent characteristics are reported in Table 2. The age category with most respondents was 30 to 59 years of age (47.1%). The respondents consisted on average of people from high income households (40.3%), with mostly upper secondary or vocational college education (49.2%). The most common region of residency of the respondents was the Helsinki-Uusimaa region (30.9%).

**Table 2:** Respondent characteristics

	% of total sample (*n* = 3244)		% of CIH users (*n* = 1657)	% of Non-CIH users (*n* = 1587)
	Unweighted	Weighted	Weighted	Weighted
Gender				
Women	52.0	50.8	58.5	42.6
Men	48.0	49.2	41.5	57.4
Age in Years				
Mean age in years (SD)	52.8 (17.8)	50.3 (18.7)	46.3 (18.3)	54.5 (18.2)
Age Groups				
16–29 years	12.0	17.0	22.2	11.7
30–59 years	46.1	47.1	50.5	43.5
60 years and above	41.9	35.9	27.4	44.8
Household Income				
Low	26.6	27.7	29.2	26.4
Middle	17.4	17.3	16.8	17.4
High	41.2	40.3	39.2	41.3
I do not know/want to answer	14.7	14.7	14.7	14.9
Highest Obtained Education ^a^				
Primary and lower secondary school	8.4	8.9	6.9	10.7
Upper secondary and vocational college education	48.8	49.2	49.6	48.7
Bachelor’s degree or higher vocational degree	23.2	23.4	25.0	21.9
Master’s degree or higher	19.1	18.5	18.4	18.7
Region of Residency				
Helsinki-Uusimaa	32.6	30.9	31.0	30.8
South Finland	22.4	25.0	19.2	23.0
West Finland	23.5	21.1	25.6	24.4
North and East Finland	21.5	23.0	24.3	21.8

^a^ 20 respondents (0.6%) did not report their educational information

### Use of CIH

Just over a half (51.1%; 95%CI: 49.4–52.8) of the respondents had used at least one CIH modality in the 12 months prior to taking the survey (Table 3). A fifth of the respondents had visited a CIH provider (20.4%, 95%CI: 19.0–21.8), whereas over a quarter of the total amount of respondents had used either CIH natural remedies (27.0%, 95%CI: 25.5–28.6) or CIH self-help practices (28.8%, 95%CI: 27.3–30.4) in the 12 months prior to the survey.

**Table 3:** Total CIH use and the usage of providers, natural remedies and self-help modalities (*n* = 3244)

	Visited/used in last 12 months (%) [95% CI]		
	total *n* = 3244	Women *n* = 1647	Men *n* = 1597
Total CIH use	51.1 [49.4, 52.8]	58.9 [56.5, 61.3]	43.0 [40.6, 45.5]
All CIH providers	20.4 [19.0, 21.8]	23.2 [21.2, 25.3]	17.5 [15.7, 19.4]
All CIH providers combined with massage therapist (conventional ^a^)	40.8 [39.1, 42.5]	47.1 [44.7, 49.5]	34.3 [32.0, 36.6]
All CIH natural remedies	27.0 [25.5, 28.6]	28.6 [26.4, 30.8]	25.4 [23.3, 27.6]
All natural remedies ^b^	81.7 [80.3, 83.0]	87.6 [85.9, 89.1]	75.6 [73.5, 77.7]
All CIH self-help practices	28.8 [27.3, 30.4]	35.9 [33.6, 38.2]	21.6 [19.6, 23.7]
All CIH self-help practices combined with praying, sauna, art, nature and other	73.0 [71.4, 74.5]	77.7 [75.6, 79.6]	68.1 [65.8, 70.4]

^a^ Not considered CIH in Finland

^b^ Including vitamins and minerals

A larger portion of women (58.9%, 95%CI: 56.5–61.3) had used CIH compared to men (43.0%, 95%CI: 40.6–45.5). Women had more often visited a CIH provider (23.2%, 95%CI: 21.2–25.3) than men (17.5%, 95%CI: 15.7–19.4), and women had more often used CIH self-help practices (35.9%, 95%CI 33.6–38.2) than men (21.6%, 95%CI: 19.6–23.7). There was no significant difference in usage of CIH natural remedies between women (28.6%, 95%CI: 26.4–30.8) and men (25.4%, 95%CI: 23.3–27.6).

### Visits to providers

**Fig. 1:**
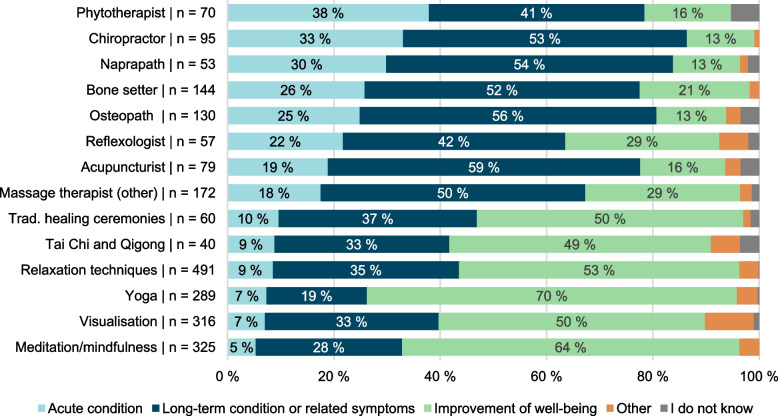
Most important reasons for latest use of selected* CIH providers and CIH self-help practices**. *CIH providers and CIH self-help practices with users n ≥ 40. **Data described in more detail in Supplementary Information

The most commonly visited CIH providers were a non-traditional/conventional massage therapist (5.3%), a bone setter (4.4%) and an osteopath (4.0%). Part of the respondents (20.6%) reported not visiting any of the providers (CIH or non-CIH) listed in the 12 months prior taking part in the survey. Over half (62.0%) of respondents who had visited a CIH provider had also visited a physician in the 12 months prior to taking the survey. Most participants reported visiting CIH providers as very or somewhat helpful (72.8–92.9%) (Table 4). The most important reason for the latest visit to CIH providers was often reported to get help for a long-term illness or condition or related symptom (Fig. 1).

**Table 4:** Visits to healthcare providers and reported helpfulness of the services

Providers	Visited in the last 12 months (%)			Visited in the last 12 months (n)	Users who found very helpful or somewhat helpful (%)	Mean/median of times visited in 3 months prior to survey (range)
	total *n* = 3244	Women *n* = 1647	Men *n* = 1597	weighted (unweighted)		
Physician ^a^	64.1	66.4	61.8	2080 (2094)	93.9	1.38/1 (0 to 20)
Chiropractor	2.9	2.4	3.5	95 (91)	90.9	1.38/1 (0 to 10)
Homeopath	1.2	1.0	1.3	38 (32)	92.9	1.42/1 (0 to 5)
Acupuncturist	2.4	2.8	2.1	79 (73)	86.5	1.34/1 (0 to 6)
Phytotherapist	2.2	1.3	3.0	70 (59)	91.6	2.43/1 (0 to 30)
Bone setter	4.4	5.7	3.1	144 (139)	86.7	1.13/1 (0 to 9)
Energy healer	1.0	1.3	0.7	32 (32)	81.7	2.17/1 (0 to 10)
Reflexologist	1.8	2.3	1.2	57 (51)	80.8	1.58/1 (0 to 12)
Aromatherapist	0.9	0.6	1.3	30 (24)	90.9	1.77/2 (0 to 6)
Massage therapist (conventional) ^a^	29.3	35.0	23.4	951 (953)	97.3	1.87/1 (0 to 35)
Massage therapist (other)	5.3	7.1	3.5	172 (170)	90.8	1.61/1 (0 to 10)
Naprapath	1.6	1.8	1.5	53 (49)	80.0	1.44/1 (0 to 8)
Osteopath	4.0	4.4	3.6	130 (120)	86.5	1.08/1 (0 to 7)
Art therapist	0.9	0.7	1.1	29 (21)	80.9	2.40/1 (0 to 9)
Cuppist	0.7	0.2	1.2	23 (17)	75.6	1.41/1 (0 to 4)
Hypnotherapist	0.7	0.1	1.4	24 (17)	83.9	1.34/1 (0 to 3)
TCM practitioner ^b^	0.8	0.7	0.9	27 (23)	78.9	1.45/1 (0 to 4)
Anthroposophic therapist	0.5	0.1	0.9	15 (10)	72.8	2.61/1 (1 to 6)
Ayurvedic practitioner	0.3	0.3	0.4	11 (9)	89.7	2.32/1 (1 to 10)
Other, what?	0.7	1.1	0.4	24 (22)	84.1	1.47/1 (0 to 4)
None mentioned above	20.6	17.6	23.6	667 (670)		
I do not know/want to say	0.6	0.2	1.0	20 (16)		

^a^ Not considered CIH in Finland

^b^ TCM Traditional Chinese Medicine

### Use of natural remedies

Vitamins and minerals (76.5%) were the most used modality, other supplements (23.5%) being the second most used. A part (17.4%) of the respondents reported not having used any natural remedies in the 12 months prior to the survey (Table 5).

**Table 5:** Usage of natural remedies (*n* = 3244)

Natural remedies	Used in last 12 months (%)			Used in last 12 months (n)
				weighted (unweighted)
	total *n* = 3244	Women *n* = 1647	Men *n* = 1597	
Vitamins and minerals ^a^	76.5	84.6	68.1	2481 (2489)
Other supplements	23.5	25.1	21.9	764 (733)
Herbs and herbal medicine	5.0	5.4	4.6	163 (159)
Homoeopathic remedies	2.2	2.5	1.9	71 (65)
Other	1.5	1.2	1.9	50 (48)
I have not used natural remedies	17.4	12.0	23.0	565 (587)
I do not know/want to say	0.9	0.4	1.3	28 (21)

^a^ Not considered CIH in the study

### Use of CIH self-help practices

Relaxation techniques (15.1%), meditation and mindfulness (10.0%), visualisation (9.7%) and yoga (8.9%) were the most used CIH self-help practices. A fourth (25.4%) of all respondents reported not having used any of the listed self-help modalities (CIH or non-CIH) in the 12 months prior to the survey. Majority of respondents reported CIH self-help practices as very or somewhat helpful (79.9–94.9%) (Table 6). The most important reason for the latest time of using a CIH self-help practice was often reported to be improvement of well-being (Fig. 1).

**Table 6:** Usage of CIH self-help practices and the reported helpfulness (*n* = 3244)

Self-help practices	Used in last 12 months (%)			Used in last 12 months (n)	Users who found very helpful or somewhat helpful (%) total	Mean/median of times practised in 3 months prior to survey (range)
	total *n* = 3244	Women *n* = 1647	Men *n* = 1597	weighted (unweighted)		
Meditation and mindfulness	10.0	13.8	6.1	325 (307)	89.4	20.1/8 (0 to 300)
Yoga	8.9	12.4	5.3	289 (277)	93.5	11.5/6 (0 to 105)
Tai Chi and Qigong	1.2	0.9	1.6	40 (33)	82.9	15.0/6 (0 to 100)
Relaxation techniques	15.1	20.6	9.5	491 (469)	94.8	16.3/6 (0 to 180)
Visualisation	9.7	12.6	6.8	316 (301)	89.8	15.9/5 (0 to 600)
Attending traditional healing ceremonies	1.8	1.9	1.7	60 (53)	79.7	3.45/1 (0 to 22)
NLP ^a^	1.1	1.2	1.1	37 (32)	94.9	3.0/2.48 (0 to 10)
Other	3.5	4.4	2.52.5	114 (120)	91.9	24.5/10 (0 to 200)
None of the above	25.4	21.2	29.7	823 (855)		
I do not know/want to say	1.5	1.1	1.9	48 (48)		

^a^ Neurolinguistic programming

### Prayer, sauna, art and nature as a form of self-help

Prayer for one’s health (11.6%), sauna (52.7%), art (20.5%) and nature (39.6%) as self-help practices, albeit not regarded as CIH in Finland, were included in the survey (Table 7). When prayer, sauna, art and nature were combined with the surveyed CIH self-help modalities, 73.0% of the respondents had used at least one modality in the 12 months prior to the survey (Table 3).

**Table 7:** Usage of prayer, sauna, art and nature and the reported helpfulness (*n* = 3244)

Self-help practice	Used in last 12 months (%) Total (women/men)			Used in last 12 months (n)	Found very helpful or somewhat helpful (%) total	Mean/median of times practised in 3 months prior to survey (range)
	total *n* = 3244	Women *n* = 1647	Men *n* = 1597	weighted (unweighted)		
Praying for one’s health	11.6	14.1	9.0	377 (378)	75.1	40.7/12 (0 to 200)
Sauna	52.7	52.8	52.5	1708 (1690)	88.0	12.9/9 (0 to 150)
Art (music, dance, literature or visual arts)	20.5	27.0	13.8	666 (647)	93.8	38.1/12 (0 to 900)
Nature ^a^	39.6	47.2	31.7	1284 (1290)	94.5	24.5/10 (0 to 300)

^a^ For calculations, one respondent was removed due to suspected error

### Intention to use

Out of the total respondents, 42.0% intended and 41.2% did not intend to use CIH in the future. Rest of the respondents did not know or want to answer (16.8%).

### Effects of Covid-19 pandemic

Out of the respondents who had visited a CIH provider (*n* = 661) in the 12 months prior to the survey, 57.8% reported that the Covid-19 pandemic had neither increased nor decreased their rate of visiting a CIH provider. Less respondents reported large increases (1.9%) or some increases (7.0%) compared to respondents who reported large decreases (10.7%) or some decreases (19.1%). A portion (3.5%) of the respondents did not know or did not want to answer.

Similar question was asked from the respondents who had used any natural remedies or self-help practices (*n* = 2650) in the 12 months prior to the survey. Most of the respondents (75%) reported that their usage of natural remedies or self-help practices neither increased nor decreased due to the Covid-19 pandemic. Out of the same group of respondents, 2.0% responded that their usage increased a lot, and 10.6% responded that their usage somewhat increased. Usage decreased a lot or somewhat decreased according to 3.3% and 5.6% of the respondents, respectively. A portion of the respondents (3.6%) did not know or did not want to answer.

## Discussion

The study examined the prevalence of CIH usage in the general Finnish population. The aim was to create knowledge about CIH use for both national and international scientific and public health communities by using an internationally recognised measurement tool. Approximately half (51.1%) of respondents in our study had used at least one CIH modality at least once in the 12 months before the survey. Women were more likely to report to having used CIH compared to men, and CIH users seemed on average younger than non-users.

The most often reported CIH modalities used by the respondents in our study were self-help practices, which were used by more than every fourth respondent (28.8%). The most often reported modalities used within the CIH self-help category were relaxation techniques (15.1%), mindfulness and meditation (10.0%), visualisation (9.7%) and yoga (8.9%). Approximately every fifth respondent reported visits to a CIH provider (20.4%). CIH natural remedies (with vitamins and minerals excluded) were used by 27.0% of the respondents. When vitamins and minerals were included in the prevalence estimate, a great majority of the respondents had used natural remedies (81.7%).

Our study has a different estimate for prevalence of CIH usage in Finland compared to the estimates of earlier studies [4, 25]. This variability between the studies is likely due to methodological differences, rather than an actual change in CIH usage. Vuolanto et al. [25] found a prevalence of 82.6% for lifetime usage of CIH in Finland. However this estimate pertained to both self and family use of CIH, making it difficult to make inferences on usage of single individuals. Additionally, they included a different set of CIH modalities compared to the one’s included in our survey. For example, Vuolanto et al. [25] did not include bone setting in their list of CIH modalities, whereas the FMA [26] included it with the result of the lifetime prevalence of 17% for bone setting. In our study 4.4% (Table 4) of respondents reported having visited a bone setter in the 12 months prior to the survey. In a study by Kemppainen et al. [4], the estimated prevalence of CIH usage in Finland (35.3%) was similarly based on a different list of included CIH modalities. For example, conventional massage therapy and physiotherapy were considered as CIH contrary to our survey.

The variation in the included treatments and practices between studies express what is defined as CIH in different countries and at different timepoints. For example, conventional massage therapists are not considered CIH providers in Finland. Almost a third (29.3%) of the respondents in our study had visited a conventional massage therapist in the year prior to the survey. Including visits to a conventional massage therapist into the final estimate of CIH usage would have increased the proportion of reported CIH users. We also included modalities such as using prayer, sauna, art and nature as self-help practices into the surveyed items, yet did not include them into the prevalence estimates of CIH use as their CIH status is not clear. Use of these modalities was generally common within our respondents (11.6–52.7%). This possibly indicates that for many participants they constitute a part of their individual health promotion toolkit.

We used a similar measurement tool (I-CAM-Q) as was used in a prevalence study conducted in Norway by Kristoffersen et al. [5]. They found a prevalence of 62.2% for overall CIH usage in Norway, including in their definition of a CIH user visits to a CIH provider, intake of natural remedies and CIH self-help practices. We attempted to define CIH usage as similarly to their study as possible in order to facilitate inter-country comparisons on CIH usage. The overall prevalence for CIH usage in the Norwegian study, when vitamins and minerals were excluded from their analysis, was 42.9% [5]. By using a comparable definition of CIH users, we found a prevalence of 51.1%.

The prevalence estimates for CIH usage in Norway and Finland differed slightly in terms for visiting a CIH provider (Norway 14.7% vs. Finland 20.4%). However, the estimates were fairly similar when chiropractors were included into the Norwegian prevalence for visiting a CIH provider (22.2%) [4]. For using self-help practices (Norway 29.1% vs. Finland 28.8%) the prevalence estimates were highly aligned. Natural remedies were used by 27.0% of the respondents in our study. In the Kristoffersen et al. [5] study, the estimate for usage of natural remedies (47.7%) included vitamins and minerals, only excluding multivitamins, possibly leading to a higher estimate compared to our study.

In regards to other Nordic countries, I-CAM-Q has been used also in Sweden. A study conducted in Southern Sweden showed higher prevalence estimates for over-all CIH usage (71%) compared to the current study [6]. Some of their estimates were similar to our study, as out of the Swedish respondents 33% had visited a CIH provider and 32% had used CIH self-help practices. For the usage of natural remedies, their estimate (53%) was noticeably higher compared to the one in the current study. However, they included vitamins and minerals into the analyses of natural remedies, which is a possible contributor to the differences seen in the prevalence estimates of natural remedy and over-all CIH usage. These studies together increase the current knowledge on CIH usage in the three studied Nordic countries. The prevalence of usage differed between individual modalities, yet the studies showed some similarities in CIH usage. For example, providers offering manipulative treatments (massage, chiropractic treatment, naprapathy, osteopathy) were among the most commonly visited CIH providers in all of the studies. Additionally, the CIH modalities used were generally experienced as helpful by users in all three studies [5, 6].

Since the time frame of our survey coincided with the ongoing Covid-19 pandemic (2020–2022), it is possible that it had an influence on the use of CIH in Finland. Most of the CIH users (57.8%) in our study reported that their visits to CIH providers neither increased nor decreased due to the Covid-19 pandemic, indicating that the pandemic did not significantly alter the rate of CIH usage in Finland. This was also found in regards to the usage of natural remedies and self-help practices (75.0%). More respondents indicated that their visits to CIH providers decreased or somewhat decreased (29.8%), compared to natural products and self-help methods (8.8%). These results point partially in the same direction as the previous Norwegian study investigating the use of CIH in connection with the Covid-19 pandemic [39].

The study has some limitations, which might affect its results and comparability. First, the response rate of 28,19% was low, which may challenge the generalizability of the findings. To increase the generalisability, the data used for the analysis were weighted by age, gender and residential area. Second, changes were made to the original version of the measurement tool, I-CAM-QN, when translating it to Finnish. Some modalities were added, and some removed from the list of CIH providers, due to cultural differences. We also included an open ended question for the “Other, what?” item in the list of CIH providers and self-help practices included in the study. The answers for the question in regards to self-help practices (*n* = 130) were too broad in scope to be analysed meaningfully in this study, and thus not included in the final estimates of CIH usage. All these changes, albeit made for the survey to better suit the Finnish public health field, might have influenced the prevalence estimates. Additionally, it should be noted that the subjective answers of the respondents’ could be influenced by recall bias.

The variation in the answers to the open-ended questions mentioned above indicate that the public opinion on CIH and public understanding on what is considered as CIH among the population greatly differs between individuals. For example, physiotherapist, nurse and psychotherapist were all recurring answers in the open ended “other” category for visited therapists in our study, even as they are not considered CIH in the Finnish healthcare system. This might have been due to the formulation of the question. Respondents might have understood that they were supposed to name all healthcare practitioners they had visited in the past 12 months, and not exclusively name CIH providers as was intended.

It seems that what is defined as CIH is dynamic and the concept of CIH is changing over time in society. Moreover, the different views expressed in public by key health policy actors in Finland may influence how CIH is understood differently in various population groups as well. For example the FMA publicly names chiropractors, osteopaths and naprapaths as “alternative therapists” [26], while these practitioners are incorporated into the national health practitioner registration system [30]. This may cause difficulties in the general public in distinguishing the line between alternative and conventional healthcare.

### Implementation of the findings

The findings provide public health policy makers with research based information on prevalence, reason to use and experienced helpfulness of a number of CIH modalities used in Finland. The findings may be utilised for education of health care personnel and the general public.

### Further research

The results indicate that the majority of CIH users in Finland consider CIH use helpful, and seemingly in particular in relation to long-term health conditions and related symptoms as well as in enhancement of well-being. Our study shows that about two thirds of respondents (64.1%) had visited a physician and nearly one third (29.3%) a massage therapist. As shown by Vuolanto et al. [24], it seems that in Finland people use different CIH modalities alongside the conventional health services, not as alternatives for them. Therefore, information is needed about the advantages and disadvantages experienced by CIH users of the combined use of CIH and conventional health services. It is also of paramount importance to further study how the evidence based CIH modalities could be integrated into the existing health promotion practices in Finland. Further research is also needed to study CIH use in disease-specific populations and to explore experienced and assumed harms caused by different CIH modalities for their users.

## Conclusions

Based on our results, CIH in its many modalities was found to be used by over a half of the Finnish population. The most common forms of CIH in Finland according to our findings were the usage of self-help practices, such as relaxation techniques, mindfulness and meditation, visualisation and yoga. The study confirms that CIH is a selection of diverse modalities of everyday health care, healing and health promotion. As most of the modalities listed in this study were experienced helpful by the CIH users, health policy makers should assess how to maximise experienced benefits and minimise potential harms of these modalities.

### Supplementary Information

**Additional file 1: Supplementary Table 1.** Most important reason for the latest visit to a provider.** Supplementary Table 2.** Most important reason for the latest use of CIH self-help practice.** Supplementary Table 3.** Most important reason for the latest use of other self-help practice.

## Data Availability

The dataset this paper has been based on has not been deposited in any repository. All dataset and materials are available from the corresponding author upon reasonable request.

## Abbreviations

CIH: Complementary and Integrative Health
CAM: Complementary and Alternative Medicine
NLP: Neuro-Linguistic Programming
TCM: Traditional Chinese Medicine
