# Supplementary material for: Use of complementary and integrative health in Finland: a cross-sectional survey
Source: BMC Complement Med Ther. 2023 Aug 4;23:279. doi: 10.1186/s12906-023-04088-4 (PMC10401804; doi:10.1186/s12906-023-04088-4)
Supplement: Supplementary file 1 — Additional file 1: Supplementary Table 1. Most important reason for the latest visit to a provider. Supplementary Table 2. Most important reason for the latest use of CIH self-help practice. Supplementary Table 3. Most important reason for the latest use of other self-help practice. [file 12906_2023_4088_MOESM1_ESM.docx]

**Supplementary tables for Figure 1 in *Use of complementary and integrative health (CIH) in Finland: cross-sectional survey***

**Supplementary Table 1**: Most important reason for the latest visit to a provider.

| Provider | Visited in last 12 months (n) | Reason for use (%) | | | | |
| --- | --- | --- | --- | --- | --- | --- |
|  |  | Acute illness | Long-term illness | Improvement of well-being | Other | I do not know |
|  | weighted (unweighted) |  |  |  |  |  |
| Physician | 2080 (2094) | 32.7 | 53.8 | 5.4 | 7.7 | 0.4 |
| Chiropractor | 95 (91) | 33.0 | 53.4 | 12.7 | 0.9 | - |
| Homeopath | 38 (32) | 31.5 | 50.3 | 8.2 | 4.6 | 5.3 |
| Acupuncturist | 79 (73) | 18.9 | 58.8 | 16.0 | 2.9 | 3.5 |
| Phytotherapist | 70 (59) | 37.9 | 40.6 | 16.3 | - | 5.3 |
| Bone setter | 144 (139) | 25.8 | 51.7 | 20.7 | 1.8 | - |
| Energy healer | 32 (32) | 13.5 | 20.1 | 46.6 | 8.8 | 11.0 |
| Reflexologist | 57 (51) | 21.7 | 41.8 | 29.0 | 5.5 | 2.0 |
| Aromatherapist | 30 (24) | 28.1 | 35.6 | 36.0 | - | - |
| Massage therapist (conventional)* | 951 (953) | 16.7 | 39.4 | 41.6 | 2.1 | 0.1 |
| Massage therapist (other) | 172 (170) | 17.5 | 49.8 | 29.1 | 2.2 | 1.4 |
| Naprapath | 53 (49) | 29.8 | 54.0 | 12.6 | 1.5 | 2.1 |
| Osteopath | 130 (120) | 24.8 | 55.8 | 13.1 | 2.7 | 3.5 |
| Art therapist | 29 (21) | 29.3 | 37.4 | 33.4 | - | - |
| Cuppist | 23 (17) | 27.2 | 17.0 | 46.7 | - | 9.0 |
| Hypnotherapist | 24 (17) | 36.6 | 42.0 | 21.4 | - | - |
| TCM practitioner** | 27 (23) | 15.5 | 64.3 | 20.3 | - | - |
| Anthroposophic therapist | 15 (10) | 30.2 | 44.3 | 18.2 | - | 7.4 |
| Ayurvedic practitioner | 11 (9) | 17.4 | 37.2 | 36.5 | 8.9 | - |
| Other, what? | 24 (22) | 6.9 | 25.2 | 48.0 | 9.0 | 10.9 |

*not considered CIH in Finland

** Traditional Chinese Medicine

**Supplementary Table 2**: Most important reason for the latest use of CIH self-help practice.

| Self-help practice | Used in last 12 months (n) | Reason for use (%) | | | | |
| --- | --- | --- | --- | --- | --- | --- |
|  |  | Acute illness | Long-term illness | Improvement of well-being | Other | I do not know |
|  |  |  |  |  |  |  |
|  | weighted (unweighted) |  |  |  |  |  |
| Meditation and mindfulness | 325 (307) | 5.3 | 27.5 | 63.5 | 3.7 | - |
| Yoga | 289 (277) | 7.3 | 18.9 | 69.6 | 3.9 | 0.3 |
| Tai Chi and Qigong | 40 (33) | 8.8 | 32.9 | 49.2 | 5.5 | 3.6 |
| Relaxation techniques | 491 (469) | 8.5 | 35.0 | 52.7 | 3.6 | 0.1 |
| Visualisation | 316 (301) | 7.0 | 32.7 | 50.1 | 9.2 | 1.0 |
| Trad. healing ceremonies | 60 (53) | 9.6 | 37.3 | 50.1 | 1.4 | 1.6 |
| NLP* | 37 (32) | 21.0 | 29.3 | 49.7 | - | - |
| Other | 114 (120) | 4.9 | 37.0 | 47.7 | 4.3 | 6.1 |

*Neurolinguistic Programming

**Supplementary Table 3:** Most important reason for the latest use of other self-help practice.

| Self-help practice | Used in last 12 months (n) | Reason for use (%) | | | | |
| --- | --- | --- | --- | --- | --- | --- |
|  |  | Acute illness | Long-term illness | Improvement of well-being | Other | I do not know |
|  |  |  |  |  |  |  |
|  | weighted (unweighted) |  |  |  |  |  |
| Praying for one’s health (377) | 377 (378) | 16.0 | 46.2 | 29.5 | 6.7 | 1.6 |
| Sauna (1708) | 1708 (1690) | 6.9 | 10.8 | 75.8 | 3.6 | 2.9 |
| Art (666) | 666 (647) | 2.2 | 11.9 | 79.7 | 3.4 | 2.7 |
| Nature (1284) | 1284 (1290) | 3.5 | 12.5 | 79.3 | 2.0 | 2.7 |
